# Δ^8^-THC Induces Up-Regulation of Glutamatergic Pathway Genes in Differentiated SH-SY5Y: A Transcriptomic Study

**DOI:** 10.3390/ijms24119486

**Published:** 2023-05-30

**Authors:** Ivan Anchesi, Giovanni Schepici, Luigi Chiricosta, Agnese Gugliandolo, Stefano Salamone, Diego Caprioglio, Federica Pollastro, Emanuela Mazzon

**Affiliations:** 1 IRCCS Centro Neurolesi “Bonino-Pulejo”, Via Provinciale Palermo, Contrada Casazza, 98124 Messina, Italy; ivan.anchesi@irccsme.it (I.A.); giovanni.schepici@irccsme.it (G.S.); luigi.chiricosta@irccsme.it (L.C.); agnese.gugliandolo@irccsme.it (A.G.); 2 Department of Pharmaceutical Sciences, University of Eastern Piedmont, Largo Donegani 2, 28100 Novara, Italy; salamone.ste@gmail.com (S.S.); diego.caprioglio@uniupo.it (D.C.); federica.pollastro@uniupo.it (F.P.); 3 PlantaChem S.r.l.s., Via Amico Canobio 4/6, 28100 Novara, Italy

**Keywords:** delta-8-tetrahydrocannabinol, SH-SY5Y cells, transcriptomic analysis, glutamatergic synapses, cholinergic synapses, dopaminergic synapses, GABAergic synapses

## Abstract

Cannabinoids, natural or synthetic, have antidepressant, anxiolytic, anticonvulsant, and anti-psychotic properties. Cannabidiol (CBD) and delta-9-tetrahydrocannabinol (Δ^9^-THC) are the most studied cannabinoids, but recently, attention has turned towards minor cannabinoids. Delta-8-tetrahydrocannabinol (Δ^8^-THC), an isomer of Δ^9^-THC, is a compound for which, to date, there is no evidence of its role in the modulation of synaptic pathways. The aim of our work was to evaluate the effects of Δ^8^-THC on differentiated SH-SY5Y human neuroblastoma cells. Using next generation sequencing (NGS), we investigated whether Δ^8^-THC could modify the transcriptomic profile of genes involved in synapse functions. Our results showed that Δ^8^-THC upregulates the expression of genes involved in the glutamatergic pathway and inhibits gene expression at cholinergic synapses. Conversely, Δ^8^-THC did not modify the transcriptomic profile of genes involved in the GABAergic and dopaminergic pathways.

## 1. Introduction

*Cannabis sativa* L. is a dioecious plant belonging to the Cannabaceae family known for its beneficial properties, even if some of its phytocompounds have a psychoactive ac-tion [1,2]. The chemical composition of its phytocompounds can be summarized in carbohydrates, terpenes, amides, amines, phytosterols, fatty acids and their esters, but the most famous are certainly cannabinoids [3].

Several studies have demonstrated the efficacy of cannabinoids in psychiatric and neurodegenerative diseases [1,2].

The most studied cannabinoid is delta-9-tetrahydrocannabinol (Δ^9^-THC), but the side effects of being psychoactive have led to its limited use. For this reason, researchers have focused their attention on cannabinoids with minor psychoactive effects such as delta-8-tetrahydrocannabinol (Δ^8^-THC). Δ^8^-THC is a double bond isomer of Δ^9^-THC. It derives from the cyclization of cannabidiol (CBD), differing from Δ^9^-THC for the position of the double bond [4,5].

It has been widely reported that cannabinoids exert their effects by targeting different receptors such as cannabinoid receptor 1 (CB_1_) and cannabinoid receptor 2 (CB_2_) included in the endocannabinoid system (ECS) [4].

Both Δ^8^-THC and Δ^9^-THC are reported to be partial agonists of CB_1_. However, the lower affinity of Δ^8^-THC for CB_1_ could explain the less psychotropic effect of Δ^8^-THC [5].

∆^8^-THC may be associated with beneficial effects, including decreased adverse effects of chemotherapy, decreased tumor cell proliferation, and analgesic properties [6].

Furthermore, in a previous study, our group demonstrated that Δ^8^-THC can exert protective effects against amyloid-β toxicity in an in vitro model of Alzheimer’s disease [7].

Cannabinoid receptors (CBs) belong to the G protein-coupled receptor family and are widely distributed in the central and peripheral nervous systems. Cannabinoids are compounds that interact with CBs; therefore, they can influence ion channels and synaptic transmission by influencing neurotransmitter release [8,9].

Synaptic transmission and plasticity are important cellular processes that enable the nervous system to process information and respond to changes in the environment and internal environment [10].

In a previous study, we showed that the phytocannabinoids CBD and Cannabigerol (CBG) are able to affect the transcription of genes involved in glutamate, GABA and do-pamine signaling [11].

Glutamate is the primary excitatory neurotransmitter of the central nervous system (CNS) and is involved in several neuronal functions including synaptic transmission, long-term potentiation (LTP), long-term depression (LTD), plasticity and excitability [12,13]. Once released into the synaptic cleft, glutamate can bind to various classes of receptors including glutamatergic receptors (GluRs), which are divided into ionotropic (iGluRs) and metabotropic (mGluRs). N-methyl-d-aspartate receptors (NMDARs), AMPA receptors (AMPARs) and kainate receptors (KARs) are classified among iGluRs that act as mediators in the fast part of excitatory transmission. Instead, mGluRs include G protein coupled receptors (GPCRs) involved both in neuronal plasticity and cognitive functions [14,15].

Through glutamate transmission, cannabinoids could indirectly act on dopaminergic signaling. Dopamine (4-(2-aminoethyl)-1,2-benzenediol) is produced by dopaminergic neurons and regulates several physiological functions such as cognition, mood, sleep, emotion, motor functions, memory and learning [16,17,18].

Cannabinoids are also able to exert their effect on cholinergic and GABAergic synapses [19,20]. Acetylcholine (ACh), one of main neurotransmitters involved in synaptic plasticity, behavior and movement, is hydrolyzed by acetylcholinesterase (AChE) present both in the synaptic cleft and neuromuscular junctions [21].

Instead, gamma-aminobutyric acid (GABA) is an inhibitory neurotransmitter that modulates synaptic transmission and neuronal development, as well as learning and depression [22,23].

The aim of this study was to investigate, using Next Generation Sequencing (NGS) technology, the transcriptomic changes on the synaptic pathways induced by Δ^8^-THC in all-trans Retinoic acid (RA)-differentiated SH-SY5Y cells.

## 2. Results

### 2.1. Differentiation of SH-SY5Y Cells with RA

**Figure 1:**
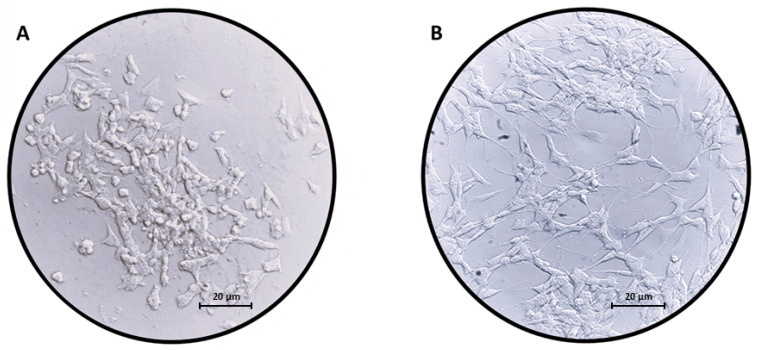
Images of (**A**) undifferentiated SH-SY5Y neuroblastoma cells and (**B**) SH-SY5Y neuroblastoma cells differentiated with RA for 5 days. The differentiation leads to changes in morphology; indeed, SH-SY5Y cells acquire a neuron-like phenotype when treated with RA. Scale bar: 20 µm.

**Figure 2:**
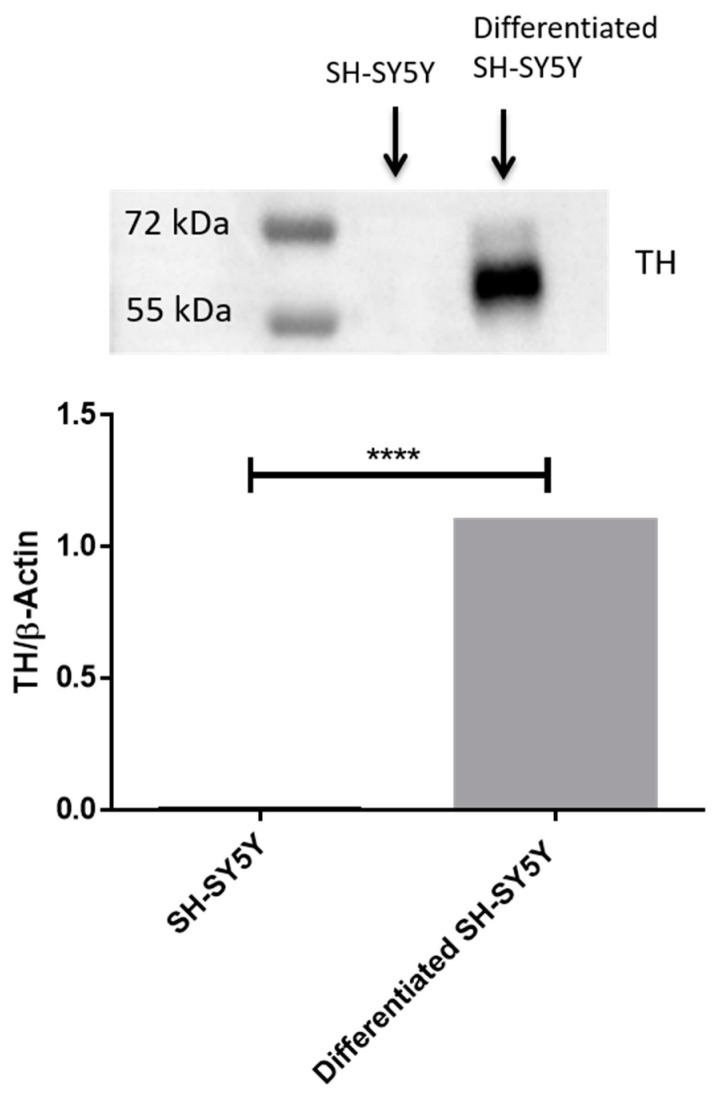
Western blot for TH. RA treatment caused an increase in TH protein levels. In order to evaluate that blots were loaded with equal amounts of protein lysates; we incubated the blot with an antibody for β-Actin. **** *p* < 0.0001. The blot for β-Actin is available in the Supplementary Figure S2.

Differentiated SH-SY5Y (Figure 1B) cells acquired a neuronal like morphology compared to undifferentiated cells (Figure 1A). Our results obtained by Western blot analysis show that only RA-differentiated SH-SY5Y cells express TH (Figure 2) compared to undifferentiated cells.

### 2.2. Transcriptomic Analysis

After treatment of differentiated SH-SY5Y cells with Δ^8^-THC, we performed a NGS transcriptomic analysis and we focused on differentially expressed genes (DEGs) between Δ^8^-THC (treated) and CTRL (non-treated) groups.

Δ^8^-THC exerts its effects on differentiated SH-SY5Y cells, as demonstrated by transcriptomic analysis. In detail, bioinformatics analysis highlighted 8819 DEGs, among which 4307 are upregulated and 4512 are downregulated DEGs. Specifically, upregulated genes are more expressed in the Δ^8^-THC group, whereas downregulated genes are more expressed in the CTRL group.

We performed a Gene Ontology analysis on the whole transcriptomic using the Panther database (http://pantherdb.org/ (accessed on 5 April 2023)), a web page that allows us to make classification and enrichment tests of DEGs.

**Figure 3:**
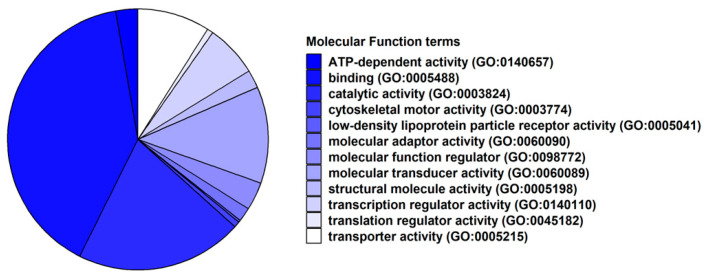
Pie chart of the classification of Gene Ontology terms related to Molecular Function. It is interesting to note that Panther classified most of the included DEGs in “ATP-dependent activity” (GO:0140657) and “binding” (GO:0005488) terms.

In Figure 3, we inspected how our DEGs are classified on the basis of their Molecular Function terms of the Gene Ontology. It is interesting to note that “ATP-dependent activity” (GO:0140657) and “binding” (GO:0005488) are the most observed terms in the classification.

**Figure 4:**
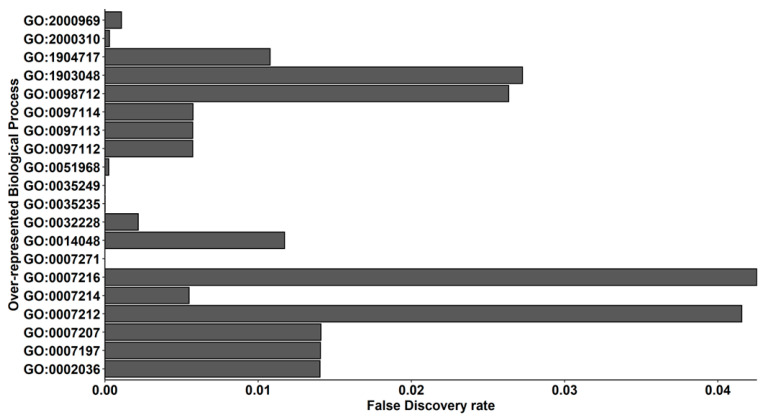
Barplot of over-represented terms in the Biological Process category of Gene Ontology related to synapses. On the x-axis, the False Discovery Rate for each term in the y-axis is represented. Even if acetylcholine (GO:1903048, GO:0007207, GO:0007197, GO:0007271) and dopaminergic (GO:0007212) terms are included, most of the terms (GO:0097114, GO:2000310, GO:0097113, GO:2000969, GO:1904717, GO:0097112, GO:0002036, GO:0035235, GO:0098712, GO:0007216, GO:0014048, GO:0051968, GO:0035249, GO:0007214, GO:0032228) are associated to glutamatergic synapsis.

In order to understand the Biological Process term in which our DEGs were expressed in, we also performed an over-representation test of our DEGs that shows the 348 terms in Table S1. Herein, the aim of our work was to observe how Δ^8^-THC is able to modulate synaptic pathways. For this reason, in Figure 4, we plotted the 20 terms that are related to synaptic activity. Of note, most terms are related to the glutamatergic pathway: “NMDA glutamate receptor clustering” (GO:0097114), “regulation of NMDA receptor activity” (GO:2000310), “AMPA glutamate receptor clustering” (GO:0097113), “positive regulation of AMPA receptor activity” (GO:2000969), “regulation of AMPA glutamate receptor clustering” (GO:1904717), “gamma-aminobutyric acid receptor clustering” (GO:0097112), “regulation of L-glutamate import across plasma membrane” (GO:0002036), “ionotropic glutamate receptor signaling pathway” (GO:0035235), “L-glutamate import across plasma membrane” (GO:0098712), “G protein-coupled glutamate receptor signaling pathway” (GO:0007216), “regulation of glutamate secretion” (GO:0014048), “positive regulation of synaptic transmission, glutamatergic” (GO:0051968), “synaptic transmission, glutamatergic” (GO:0035249), “gamma-aminobutyric acid signaling pathway” (GO:0007214), “regulation of synaptic transmission, GABAergic” (GO:0032228). Remaining terms are associated to cholinergic terms: “regulation of acetylcholine-gated cation channel activity” (GO:1903048), “phospholipase C-activating G protein-coupled acetylcholine receptor signaling pathway” (GO:0007207), “adenylate cyclase-inhibiting G protein-coupled acetylcholine receptor signaling pathway” (GO:0007197), “synaptic transmission, cholinergic” (GO:0007271). Only “dopamine receptor signaling pathway” (GO:0007212) is linked to dopaminergic terms.

**Figure 5:**
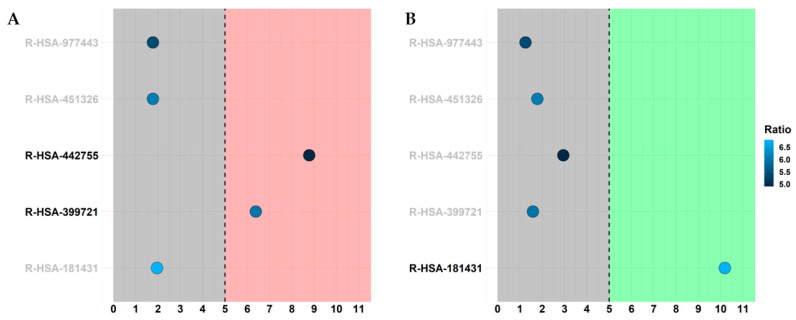
Bubbleplot of the Reactome pathways for glutamatergic, cholinergic and gabaergic synapses. The x-axis represents a score obtained by −log_2_(FDR), so that pathways with score higher than 5 are significantly upregulated (red region in panel (**A**)) or downregulated (green region in panel (**B**)). Each point is filled with the color obtained with a score computed as −log_2_(entities ratio).

Next, we took advantage of the Amigo2 project. Specifically, Amigo2 is a web page (http://amigo.geneontology.org/amigo (accessed on 5 April 2023)) that allows us to perform queries in the Gene Ontology database that, in turn, collects all the knowledge about genes characterized for biological process terms. For this reason, we used Amigo2 to retrieve all the genes that are included in the biological process term “nervous system process” (GO:0050877) and we kept all DEGs that fall in the ontology. In detail, 366 DEGs are in the GO:0050877, among which 168 are upregulated and 198 are downregulated. Thus, we searched in the Reactome database, in which the sub-pathways of “Neuronal System” (R-HSA-112316) were enriched. Indeed, Reactome returns the False Discovery Rate (FDR) for each pathway, showing the significance of the pathway itself. We found that the only enriched pathway (FDR < 0.05) was “Neurotransmitter receptors and postsynaptic signal transmission” (R-HSA-112314). Thus, we inspected all the sub-pathways: “Activation of NMDA receptors and postsynaptic events” (R-HSA-442755), “Glutamate binding, activation of AMPA receptors and synaptic plasticity” (R-HSA-399721), “Acetylcholine binding and downstream events” (R-HSA-181431), “GABA receptor activation” (R-HSA-977443), “Activation of kainate receptors upon glutamate binding” (R-HSA-451326). In detail, in both figures, each sub-pathway in the y axis is highlighted by its significance in the x-axis with a score computed by −log_2_(FDR). In this line, only the pathways in the red or green regions that have a score higher than 5 are significative because they have an FDR lower than 0.05, and they are highlighted in bold. Additionally, each pathway was filled by a color palette that shows the ratio of DEGs we found in the pathway over the total number of entities that define that pathway in Reactome. In particular, we define a score computed as -log_2_(entities ratio) so that pathways with brighter filler have more DEGs in spite of the number of entities in the pathway itself. Thus, in Figure 5A, the only enriched pathways are glutamatergic pathways related to NMDA (R-HSA-442755) and AMPA (R-HSA-399721) so that they are upregulated. In contrast, in Figure 5B, the “Acetylcholine binding and downstream event” (R-HSA-181431) pathway is the only enriched and downregulated pathway. No significance was observed for GABAergic synapses. Of note, not even pathways related to dopaminergic synapses were enriched, but it is not included in the plot because it is not included in any enriched pathway mentioned above. All the data obtained by Reactome that were plotted are in Table S2.

**Figure 6:**
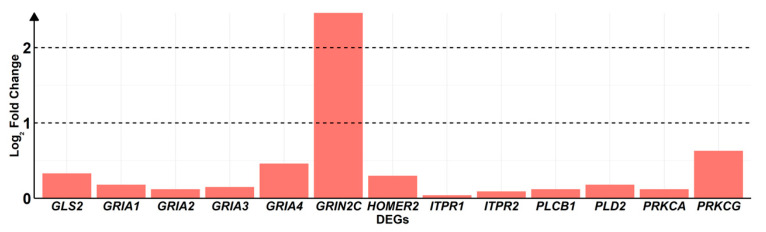
Barplot of DEGs highlighted in KEGG “Glutamatergic synapse pathway” (hsa04724). Each bar in the x-axis highlights the order of magnitude of log_2_ fold changes obtained by log_2_(∆^8^-THC/CTRL) in the y-axis. All DEGs taken into consideration are upregulated; thus, they are more expressed in ∆^8^-THC than CTRL.

Particularly, we focus our attention on the KEGG “Glutamatergic synapse pathway” (hsa04724). As shown in Figure 6, Δ^8^-THC upregulates the following 13 genes: *GLS2*, *GRIA1*, *GRIA2*, *GRIA3*, *GRIA4*, *GRIN2C*, *HOMER2*, *ITPR1*, *ITPR2*, *PLCB1*, *PLD2*, *PRKCA*, *PRKCG*.

In this line, Table 1 highlights that Δ^8^-THC is able to modulate transcripts that are associated with the translation of α-amino-3-hydroxy-5-methyl-4-isoxazolepropionic acid receptor (AMPA receptor) (*GRIA1*, *GRIA2*, *GRIA3*, *GRIA*), N-methyl-D-aspartate receptor (NMDA receptor) (*GRIN2C*), IP3R protein involved in calcium signaling (*ITPR1* and *ITPR2*), and genes involved in the downstream of metabotropic glutamate receptors (mGluR) activity (*PLCB1*, *PLD2*, *PRKCA*, *PRKCG*).

In order to highlight that the role of the glutamatergic pathway is biologically considerable in spite of gabaergic, cholinergic and dopaminergic ones, Table 2 highlights key DEGs of these synapses.

## 3. Discussion

In our study, we used RA-differentiated SH-SY5Y cells and treated them with (20 µM) Δ^8^-THC. The aim was to verify, through transcriptomic analysis, whether this cannabinoid modifies the expression of genes belonging to the synaptic pathways.

RA has powerful growth-inhibiting and cellular differentiation-promoting properties [24,25].

As shown in Figure 1, the SH-SY5Y cells treated with RA assumed a neuronal phenotype compared to the undifferentiated ones. Furthermore, to support the differentiation process, we analyzed the expression levels of the protein tyrosine hydroxylase (TH). This protein is a neuronal enzyme that catalyzes the conversion of the amino acid L-tyrosine to L-3,4-dihydroxyphenylalanine (L-DOPA) [26,27]. As shown in Figure 2 (Figure S1), TH was expressed only in RA-treated SH-SY5Y and not in the control, thus indicating that SH-SY5Y cells are differentiated. As housekeeping protein we have used β-Actin (Figure S2).

In a previous paper, we demonstrated that Δ^8^-THC showed no toxicity in a range of concentrations 5–20 µM. Moreover, the dose 20 µM Δ^8^-THC was able to restore cell viability after 10 µM Amyloid Beta treatment [7], suggesting that Δ^8^-THC may exert important neuroprotective effects. Considering this background, we studied how Δ^8^-THC affects the transcriptomic profile of genes involved in synaptic pathways, since there is no documented evidence about its role on the modulation of synaptic pathways.

Chemical signals are transferred between neurons in the nervous system through the neuro-transmitters released at presynaptic sites, followed by the diffusion across the synaptic boundaries, and the activation of post-synaptic receptors. There are several ways of communication among neurons, regulated by the release of specific neurotransmitters and subsequent responses given by their specific receptors in the post-synaptic neurons. As was previously reported in the literature, exposure to cannabinoids affects synaptic pathways, changing the outcome of various neurobiological signals [11,28,29,30,31]. For this reason, in this study, we wanted to focus our attention on the effect of Δ^8^-THC on the transcriptomic profile of genes involved in synaptic pathways related to synaptic transmission.

At first, we performed a GO analysis and classification of molecular functions and biological processes. Notably, “ATP-dependent activity” and “binding” were the most observed molecular functions (Figure 3 and Figure 4 and Tables S1 and S2). This result can be explained by the neuronal physiology, given that they represent high energy-demanding cells, consuming about 20% of the resting energy of the body.

**Figure 7:**
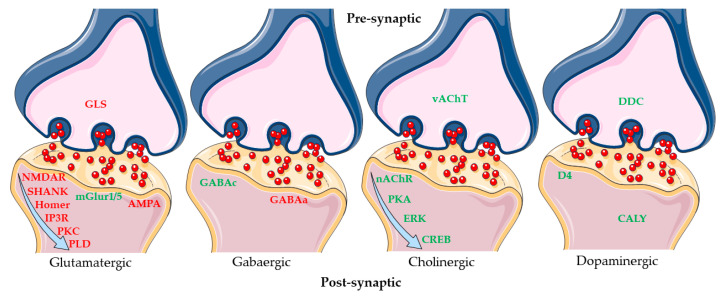
Mechanism modulated in SH-SY5Y cells differentiated with RA after Δ^8^-THC treatment. The figure represents how the different synapses are influenced by Δ^8^-THC treatment and the genes that take place. The red genes are upregulated; the green genes are downregulated. The results of transcriptomic analysis suggest that Δ^8^-THC influenced the Glutamatergic synapse more than others synapse types. The figure was created using Servier Medical Art by Servier (http://smart.servier.com/ (accessed on 9 April 2023)), licensed under a Creative Commons Attribution 3.0 Unported License (https://creativecommons.org/licenses/by/3.0/ (accessed on 9 April 2023)).

Our transcriptomic analysis demonstrated that Δ^8^-THC influenced several genes involved in “Glutamatergic Synapse” and “Cholinergic synapse”. Instead, it is not able to exert an action on “GABAergic synapse” and “Dopaminergic synapse” (Figure 7).

Glutamate and GABA are important neurotransmitters in the excitatory and inhibitory balance that neurons exhibit [32] because many aspects of the physiological brain function, such as cognition memory and learning, are directly or indirectly affected by them [18,33,34,35].

Our analysis showed that Δ^8^-THC influenced the expression of genes involved in glutamatergic pathways related to NMDA (R-HSA-442755) and AMPA (R-HSA-399721) but not in the kainate receptor (R-HSA-451326) signaling (Figure 5). In particular, Δ^8^-THC promoted the expression of several genes of Glutamatergic Synapse pathway such as *GLS2*, *GRIA1*, *GRIA2*, *GRIA3*, *GRIA4*, *GRIN2C*, *SHANK3*, *HOMER*, *ITPR1*, *ITPR2* (Table 1 and Figure 6).

Glutaminase 2, encoded by the *GLS2* gene, is an enzyme belonging to the glutaminase family. This protein catalyzes the synthesis of glutamate by converting glutamine inside the mitochondrion. Once glutamate is released from presynaptic neurons, the neuro-transmitter predominantly binds AMPA and NMDA receptors. These are the main receptors of glutamatergic synapse and they are important for plasticity and neuronal transmission [36,37]. AMPA receptors are predominantly expressed in postsynaptic neurons and can be composed by four different types of subunits (from GluA1 to GluA4) [14]. Our transcriptomic analysis showed that Δ^8^-THC induced the upregulation of gene encoding for all the subunits of AMPA receptors (*GRIA1*, *GRIA2*, *GRIA3*, *GRIA4*). The AMPA receptor is important for memory, thus the deletion of a single AMPA receptor subunit, such as GluA1, results in alteration of the short-term memory [38].

Furthermore, we have also shown that NMDA receptor subunit epsilon-3 (*GRIN2C*) expression is influenced by Δ^8^-THC. This receptor is permeable to sodium and potassium ions and also to calcium, an important second messenger, known as an activator of several forms of intracellular signaling [39]. AMPA and NMDA receptors interact with each other during neurotransmission; in fact, the NMDA channel is activated due to release of Mg^2+^ ions blockade, which occurs after cation influx into the neuron via the AMPA receptor activity [40]. Considering these results, we suppose that Δ^8^-THC could modulate the AMPA and NMDA functions in the glutamatergic activity.

Moreover, we discovered up-regulation of other important genes involved in AMPA and NMDA signaling. SHANK3 and HOMER proteins, which are respectively encoded by SHANK3 and HOMER, are scaffold proteins that permit the co-localization of AMPA and NMDA receptors in post-synaptic neurons, also known as postsynaptic density (PSD), to induce neuronal depolarization [41,42]. The *SHANK3* gene is involved in glutamatergic synaptic transmission and long-term potentiation in the hippocampus [43]. HOMER contributes to form a physical link among signaling molecules in glutamatergics. PSD also interacts with inositol triphosphate receptor (IP3R) and mGluRs [44,45]. IP3R is a receptor involved in calcium signaling, which is important for the glutamatergic synapse [46]. Interestingly, our results showed an upregulation of *ITPR1* and *ITPR2* genes, both encoding for IP3R. Indeed, SHANK3, HOMER and IP3R upregulation support the hypothesis that there is an activation of the glutamatergic synapse after Δ^8^-THC treatment.

Furthermore, another set of genes that were upregulated in our analysis are *PLCB1, PRKCA*, *PRKCG*, *PLD2* (Table 1 and Figure 6). The proteins encoded by these genes are involved in mGluRs signaling in glutamatergic synapses. In fact, when activated, the beta-type phospholipase C (PLC-β) enzymes (*PLCB1*) [47] synthetize diacyl glycerol (DAG) and inositol trisphosphate (IP3) through the hydrolyzation of phosphatidylinositol 4,5-bisphosphate (PIP2). DAG and IP3R activity are important for Protein-kinase C (PKC), encoded by *PRKCA* and *PRKCG*. PKC plays an important role in the regulation of phospholipase D (PLD) activity [48], which is encoded by *PLD2* and in turn synthesizes Phosphatidic acid (PtdOH), an anionic phospholipid involved in neuro-transmission [49]. These results also suggest that the mGluRs pathway could be influenced by Δ^8^-THC.

Furthermore, our findings suggest how Δ^8^-THC does not exert an action on the other types of synapses, or it is able to induce inhibitory effects. Unlike the GLS2 gene, our analyses do not show a deregulation of the GAD gene, encoding for glutamic acid decarboxylase (GAD), which plays a role in the synthesis of GABA from glutamate [50].

There are several GABA receptors, such as GABAA, GABAB and GABAC [51,52]. GABAA is an ionotropic receptor, composed of pentameric combinations of different subunits [53]. Using KEGG and inspecting the “Glutamatergic synapse pathway”, there are 16 genes encoding for GABAA subunits, but only two were deregulated in our analysis (*GABRA3*, *GABRG3*). On the counterpart, our analyses have highlighted that *GABRR2* gene, encoding for a subunit of GABAC, is downregulated while the genes encoding for GABAB are not influenced by Δ^8^-THC. As can be seen in Figure 5 (R-HSA-977443), these results suggest that Δ^8^-THC does not affect the GABAergic synapse pathway and that there could be a higher concentration of glutamate in treated cells than in the control. For these reasons, we suppose that Δ^8^-THC treatment could induce stronger glutamatergic action compared to the inhibitory action of GABA.

Our results suggest that Δ^8^-THC exerts an inhibitory action in “Acetylcholine binding and downstream events” (R-HSA-181431) (Figure 5). Especially, in our work, we show that Δ^8^-THC treatment inhibits the transcription of genes involved in cholinergic synapse pathways such as *SLC18A3*, *CHRNA3*, *CHRNA6*, *CHRNA7*, *CHRNB2*, *MAPK1*, *CREB3L3*, *CREB3L4*, *CREB5*, *DDC*, *DDR4* and *CALY* (Table 2).

Ach is a neurotransmitter that supports cognitive functions and activates muscle activity and contraction. Moreover, it is the major neurotransmitter in the autonomic nervous system. Neurons that are able to secrete acetylcholine are known as cholinergic [54]. Once this neurotransmitter is released into the synaptic cleft, it is able to bind the Nicotinic acetylcholine receptors (nAChRs) [55].

We noticed that the *SLC18A3* gene, which encodes for the vesicular acetylcholine transporter (VAChT), is downregulated. Its role in the presynaptic terminal is related to the transport of acetylcholine (Ach) into vesicles for eventual release into synapses [56].

Its downregulation supports the idea that there is a reduction of acetylcholine release in the synaptic cleft.

Our analysis showed that the genes encoding for nAChRs subunits (*CHRNA3*, *CHRNA6*, *CHRNA7* and *CHRNB2*) are mostly downregulated. These receptors are composed of five subunits, arranged around a water-filled pore [57]. nAChRs are permeable to small monovalent and divalent cations, such as sodium and calcium. The latter generates specific and complex signals involving adenylyl cyclase and protein kinase A (PKA) [58,59]. The subsequent activation of PKA determines the activation of ERK (*MAPK1*), which exerts downstream signaling through CREBs (*CREB3L3*, *CREB3L4*, *CREB5*). ERK plays a role in a number of cellular processes, including cholinergic synapse pathways [59,60]. The downregulation of genes encoding for these proteins suggest once again that the Δ^8^-THC treatment could have an inhibitory action on cholinergic synapse activity.

In addition, our results showed that Δ^8^-THC treatment does not have an effect on dopaminergic synapses. Dopamine is a neurotransmitter belonging to the catecholamine family. Among the functions that are controls, there are: movement, working memory, pleasure, reward, sleep regulation and cognitive functions [61]. DOPA decarboxylase (DDC) is a lyase enzyme that is able to regulate the transformation of L-DOPA to dopamine [62]. The *DDC* gene, which encodes for DDC, is downregulated in our analyses, suggesting a low dopamine production. Moreover, in order to exert its action, dopamine interacts with its receptors, which are G protein-coupled receptors [63]. Table 2 shows the DRD4 gene, encoding for Dopamine receptor D4 (DRD4), which is down-regulated, thus supporting the hypothesis that dopaminergic activity is low in treated cells. Except DRD4, in our analyses, there are no deregulations of other genes encoding for dopamine receptors. Moreover, our results show downregulation of the gene expression of CALY, encoding for Neuron-specific vesicular protein calcyon (Calcyon). Calcyon plays an important role in DRD1 signaling and it is involved in neuropsychiatric disorders such as schizophrenia, bipolar disorder and attention disorder [64]. These results suggest that Δ^8^-THC treatment does not increase dopaminergic synapse activity. Instead, it could inhibit dopaminergic synapse activity through inhibition of dopaminergic receptor expression.

In conclusion, our analysis demonstrated that Δ^8^-THC is able to influence the transcriptomic profile of genes involved in synaptic functionality.

## 4. Materials and Methods

### 4.1. Synthesis and Purification of Δ^8^-THC

All details about synthesis and purification were already published by Gugliandolo, A et al. [7]. Supplementary Materials presented all the details.

### 4.2. Cell Culture, Differentiation and Treatment with Δ^8^-THC

The SH-SY5Y cell line was acquired from American Type Culture Collection (ATCC) (Manassas, VA, USA). We grew the human neuroblastoma cell line SH-SY5Y in a monolayer at 37 °C in a 5% CO_2_ humidified atmosphere using Dulbecco’s Modified Eagle’s Medium/Nutrient Mixture F-12 Ham (DMEM/F12) medium (Sigma-Aldrich, St. Louis, MO, USA) supplemented with 10% fetal bovine serum (FBS) (Sigma-Aldrich), 1% glutamine and 1% penicillin-streptomycin (100 U-100 µg/mL). To induce neuronal differentiation of SH-SY5Y cells, we incubated them for 5 days with 10 µM of all-trans RA (Sigma-Aldrich) [7,65]. We dissolved Δ^8^-THC in DMSO and then diluted in PBS. We added it in the medium at the final concentration (the final DMSO concentration was <0.1%). We treated the differentiated SH-SY5Y cells with 20 µM Δ^8^-THC for 24 h.

### 4.3. Western Blot Analysis for Tyrosine Hydroxylase

Proteins were extracted from undifferentiated and RA-differentiated SH-SY5Y cells using RIPA Buffer and quantified using Bradford Assay (Bio-Rad, Hercules, CA, USA). After denaturation at 95 °C, 25 µg of protein for each sample was separated by SDS-polyacrylamide gel electrophoresis (SDS-PAGE) and transferred onto PVDF membranes (Immobilon–P, Millipore, Burlington, MA, USA). Membranes were blocked with 5% skimmed milk in TBS for 1 h. The blots were incubated at 4 °C overnight with Anti-Tyrosine Hydroxylase (1:1000, Millipore, Temecula, CA, USA) and Anti-β-Actin (1:1000, Santa Cruz Biotechnology, Dallas, TX, USA). The blots were incubated for 1 h at room temperature with the following secondary antibodies: mouse anti-rabbit IgG-HRP (1:850, Santa Cruz Biotechnology, Dallas, TX, USA) and Chicken anti-Mouse IgG Secondary Antibody, HRP (1:1000, ThermoFisher, Rockgord, IL, USA). ChemiDocTM MP System (Bio-Rad) acquired bands after exposure to an enhanced chemiluminescence system (Luminata Western HRP Substrates, Millipore, Burlington, MA, USA).

### 4.4. RNA Extraction and Library Preparation

Cells were harvested and RNA was extracted using Maxwell^®^ RSC simplyRNA Cells Kit (Promega, Madison, WI, USA) following the protocol. The library was prepared using the TruSeq RNA Exome protocol (Illumina, San Diego, CA, USA) following the manufacturer’s instruction. Experiments were performed in triplicate.

### 4.5. Sequencing Analysis

We used the fastqc tool version 0.11.4 from the Babraham Institute (Cambridge, UK) to evaluate the raw data obtained from the NextSeq 550 Dx instrument of Illumina. We then eliminated adapters and low-quality bases with Trimmomatic version 0.38 (Usadel Lab, Aachen, Germany) [66]. In detail, we used the paired-end option (PE), the -phred33 encoding, 2 seed mismatches, 30 palindrome clips and 10 simple clip thresholds for the ILLUMINACLIP, 20 of both for LEADING and TRAILING, 4 in window size and 15 for the quality of the window in SLIDINGWINDOW, with 75 minimum length. In the next step, we aligned the cleaned reads to the human reference genome (GRCh38) with the STAR RNA-seq aligner 2.7.3a (New York, NY, USA) [67]. We used the primary assembly annotation file v39 of gencode for -sjdbGTFfile option, the -outFilterIntronMotifs RemoveNoncanonical and the -quantMode GeneCounts. In the last step, we computed the expression levels of the transcripts with the htseq-count python package version 0.6.1p1 (European Molecular Biology Laboratory (EMBL), Heidelberg, Germany) [68] using the -s reverse option.

### 4.6. Comparative Analysis and In Silico Inspection

Once the expression was obtained, we identified differentially expressed genes (DEGs) with the DESeq2 library in R version 3.6.3 (R Core Team) [69]. The full script (script.R) is included the supplementary materials. In detail, we performed a Wald test for each gene by DESeq2, returning a *p*-value. Because of the multiple Wald tests performed, we defined the *q*-value as the *p*-value corrected by the Benjamini–Hochberg procedure to drop the number of false positives. Thus, a gene was marked as DEG only if the *q*-value was lower than 0.05. Panther web page (current release 17.0) was used to perform classification and enrichment analysis with default parameters [70]. From Amigo2, we retrieved all protein coding genes included in the “nervous system process” (GO:0050877). Then, DEGs in the GO:0050877 were enriched for pathways using Reactome web page database. In detail, we used the analysis tool including default parameters. Thus, we kept the “project to human” option, allowing us to include information related to association coming from other species about orthologous genes in the analysis; we did not include interactors. We define all pathways with a p-value corrected with the Benjamini–Hochberg post-hoc procedure lower than 0.05 as significative. Then, based on the aim of our work, we inspected the “Neurotransmitter receptors and postsynaptic signal transmission” (R-HSA-112314) pathway included in the “Neuronal System” category [71,72].

## 5. Conclusions

Our analysis shows that Δ^8^-THC is able to upregulate the expression of genes involved in glutamate signaling involving AMPA and NMDA. In parallel, treatment with Δ^8^-THC reduces the expression of genes belonging to the cholinergic synapse pathway and it do not seem to have effect on the expression of genes involved in GABAergic and dopaminergic signaling. Based on our transcriptomic results, Δ^8^-THC could be considered as a potential compound that is useful to improve glutamatergic transmission in neurons. In this regard, further studies are needed to validate these results.

## Figures and Tables

**Table 1:** Differentially expressed genes involved in KEGG Glutamatergic synapse pathway (hsa04724).

Gene	CTRL	Δ^8^-THC	log_2_ Fold Change	*q*-Value	Protein
*GLS2*	120.39	151.17	0.33	2.38 × 10^−2^	GLS
*GRIA1*	494.82	561.49	0.18	1.19 × 10^−2^	AMPA receptor
*GRIA2*	1928.70	2098.12	0.12	8.32 × 10^−4^	
*GRIA3*	1727.64	1915.39	0.15	8.36 × 10^−5^	
*GRIA4*	541.77	745.06	0.46	1.46 × 10^−13^	
*GRIN2C*	2.41	13.29	2.46	4.22 × 10^−3^	NMDA receptor
*HOMER2*	816.27	1007.53	0.30	6.95 × 10^−9^	Homer
*SHANK3*	1790.25	1910.40	0.09	1.52 × 10^−2^	SHANK
*ITPR1*	9393.09	9664.99	0.04	1.53 × 10^−2^	IP3R
*ITPR2*	1542.24	1645.44	0.09	2.57 × 10^−2^	
*PLCB1*	1167.82	1269.17	0.12	1.16 × 10^−2^	PLC
*PLD2*	1390.54	1577.33	0.18	1.25 × 10^−5^	PLD
*PRKCA*	8513.01	9226.42	0.12	1.58 × 10^−12^	PKC
*PRKCG*	87.89	136.22	0.63	6.35 × 10^−5^	

For each DEG, the difference in the level of expression computed by log_2_(∆^8^-THC/CTRL) results in the log_2_ fold change. The post hoc correction of the *p*-value is highlighted in *q*-Value column. All values were rounded to the second decimal digit.

**Table 2:** Differentially expressed genes involved in gabaergic, cholinergic or dopaminergic synapsis.

Gene	CTRL	Δ^8^-THC	log_2_ Fold Change	*q*-Value	Synapsis
*GABRA3*	551.40	616.31	0.16	2.03 × 10^−2^	Gabaergic
*GABRG3*	50.57	98.84	0.97	1.12 × 10^−6^	Gabaergic
*GABRR2*	30.10	17.44	−0.79	2.27 × 10^−2^	Gabaergic
*CHRNA3*	3521.51	3364.80	−0.07	2.01 × 10^−2^	Cholinergic
*CHRNA6*	15.65	1.66	−3.24	1.60 × 10^−4^	Cholinergic
*CHRNA7*	2073.17	1844.79	−0.17	2.26 × 10^−6^	Cholinergic
*CHRNB2*	9219.72	8367.57	−0.14	5.31 × 10^−18^	Cholinergic
*PRKACB*	3247.01	3042.53	−0.09	1.17 × 10^−3^	Cholinergic
*MAPK1*	1856.47	1716.04	−0.11	3.28 × 10^−3^	Cholinergic
*CREB3L3*	13.24	4.98	−1.41	1.67 × 10^−2^	Cholinergic
*CREB3L4*	1438.70	1281.63	−0.17	1.24 × 10^−4^	Cholinergic
*CREB5*	2677.55	2544.16	−0.07	2.34 × 10^−2^	Cholinergic
*SLC18A3*	280.52	213.47	−0.39	1.11 × 10^−4^	Cholinergic
*DRD4*	108.35	54.82	−0.98	3.20 × 10^−8^	Dopaminergic
*CALY*	7.22	1.66	−2.12	2.62 × 10^−2^	Dopaminergic
*DDC*	13,066.29	12,497.37	−0.06	4.46 × 10^−6^	Dopaminergic

For each DEG, the difference in the level of expression computed by log_2_(∆^8^-THC/CTRL) results in the log_2_ fold change. The post hoc correction of the *p*-value is highlighted in *q*-Value column. All values were rounded to the second decimal digit.

## Data Availability

The data presented in this study are openly available in the NCBI Sequence Read Archive at BioProject accession numbers PRJNA973106.

## Supplementary Materials

The supporting information can be downloaded at: https://www.mdpi.com/article/10.3390/ijms24119486/s1. References [7,73,74] are cited in Supplementary Materials.
