# Supplementary material for: Δ8-THC Induces Up-Regulation of Glutamatergic Pathway Genes in Differentiated SH-SY5Y: A Transcriptomic Study
Source: Int J Mol Sci. 2023 May 30;24(11):9486. doi: 10.3390/ijms24119486 (PMC10253367; doi:10.3390/ijms24119486)
Supplement: Supplementary file 1 [file ijms-24-09486-s001.zip › Supplementary Materials.pdf]

International Journal of
Molecular Sciences

MDPI

Article

Δ8 –THC induces up-regulation of glutamatergic pathway genes in differentiated SH-SY5Y: a transcriptomic study

4. Materials and Methods

4.1. Synthesis and Purification of Δ⁸-THC

In order to synthetize of Δ^8-THC, we added p-toluensulfonic acid (11 mg, 0.064 mmol, 0.1 eq) in solution of CBD (200 mg, 0.636 mmol, 1eq)/DCM (5 mL). The starting material was converted via reflux (6 h) and TLC (Rf = 0.67, silica, petroleum ether-EtOAc 95:5), then quenched with $NaHCO_3$ s.s. and diluted with DCM. A brine wash, drying, and evaporation were performed on the combined organic phases. The residue was purified by GCC on silica gel (pure petroleum ether to petroleum ether-EtOAc 9:1) to afford 182 mg (91%) of Δ^8-THC as a brown oil.

To the purification of latter impure Δ^8-THC we used JASCO Hichrom, 250 × 25 mm, silica UV−vis detector-2075 plus (silica, petroleum-ether-EtOAc gradient from 95:5 to 85:15) and we obtained 150 mg of Δ^8-THC (1, 99%) as a brownish powder. The compound was purified by HPLC and the purity was higher than 98%. The structure was identified using ^{1}H NMR obtaining the spectra ^{1}H NMR (400 MHz, Chloroform-d) δ 6.31 (d, *J* = 1.6 Hz, 1H), 6.13 (d, *J* = 1.6 Hz, 1H), 5.46 (d, *J* = 5.9 Hz, 1H), 4.99 (s, 1H), 3.24 (dd, *J* = 15.8, 4.3 Hz, 1H), 2.74 (td, *J* = 10.7, 4.6 Hz, 1H), 2.46 (dt, *J* = 7.4, 3.6 Hz, 2H), 2.20 – 2.14 (m, 1H), 1.91 – 1.79 (m, 3H), 1.73 (s, 3H), 1.59 (p, *J* = 7.5 Hz, 2H), 1.37 – 1.25 (m, 4H), 1.14 (s, 3H), 0.92 (t, *J* = 6.8 Hz, 3H) and it was already published by Gugliandolo, A. et al. [1]. The spectra is fully compatible to the one reported in literature and reported in the literature [2,3]. With Bruker 400 spectrometers (Bruker®, Billerica, MA, USA), ^{1}H 400 MHz NM spectra were measured. Chemical shifts were referenced to the residual solvent signal ($CDCl_3$: δH = 7.26). Silica gel 60 (70-230 mesh) used for low-pressure chromatography was purchased from Macherey-Nagel (Düren, Germany). To monitored the purifications was used TLC on Merck 60 F254 (0.25 mm) plates, visualized by staining with 5% H_2SO_4 in EtOH and heating. Chemical reagents and solvents were from Aldrich (Darmstadt, Germany) and were used without further purification unless stated otherwise. HCPL JASCO Hichrom, 250 × 25 mm, silica UV−vis detector-2075 plus (Tokyo, Japan).

References

1. Gugliandolo, A.; Blando, S.; Salamone, S.; Caprioglio, D.; Pollastro, F.; Mazzon, E.; Chiricosta, L. Delta(8)-THC Protects against Amyloid Beta Toxicity Modulating ER Stress In Vitro: A Transcriptomic Analysis. *International journal of molecular sciences* **2023**, *24*, doi:10.3390/ijms24076598.
2. Choi, Y.H.; Hazekamp, A.; Peltenburg-Looman, A.M.; Frederich, M.; Erkelens, C.; Lefeber, A.W.; Verpoorte, R. NMR assignments of the major cannabinoids and cannabiflavonoids isolated from flowers of Cannabis sativa. *Phytochemical analysis : PCA* **2004**, *15*, 345-354, doi:10.1002/pca.787.
3. Gaoni, Y.; Mechoulam, R. The isolation and structure of delta-1-tetrahydrocannabinol and other neutral cannabinoids from hashish. *J Am Chem Soc* **1971**, *93*, 217-224, doi:10.1021/ja00730a036.
