# Supplementary figures and images for: Δ8-THC Induces Up-Regulation of Glutamatergic Pathway Genes in Differentiated SH-SY5Y: A Transcriptomic Study
Source: Int J Mol Sci. 2023 May 30;24(11):9486. doi: 10.3390/ijms24119486 (PMC10253367; doi:10.3390/ijms24119486)

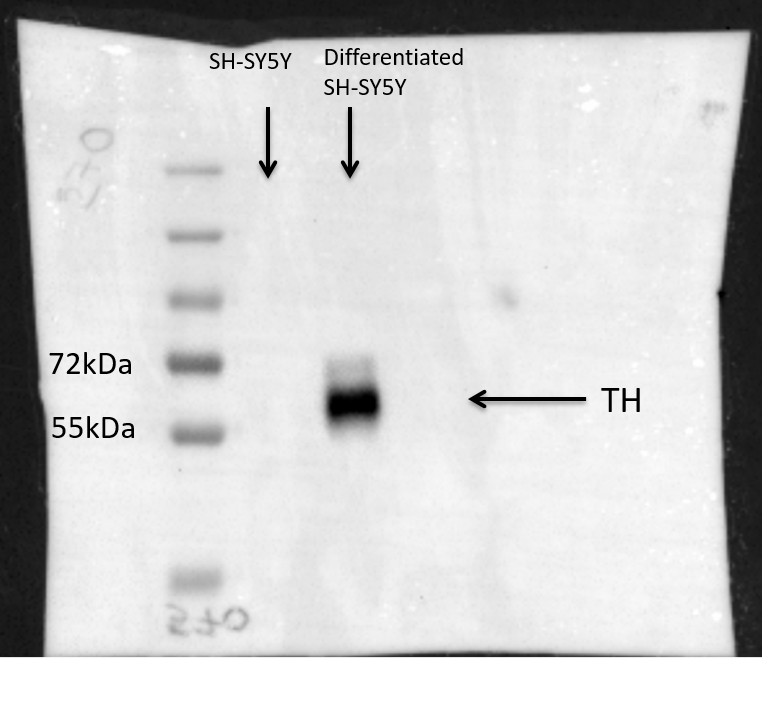

Supplement: Supplementary file 1 [file ijms-24-09486-s001.zip › Figure S1.tif]

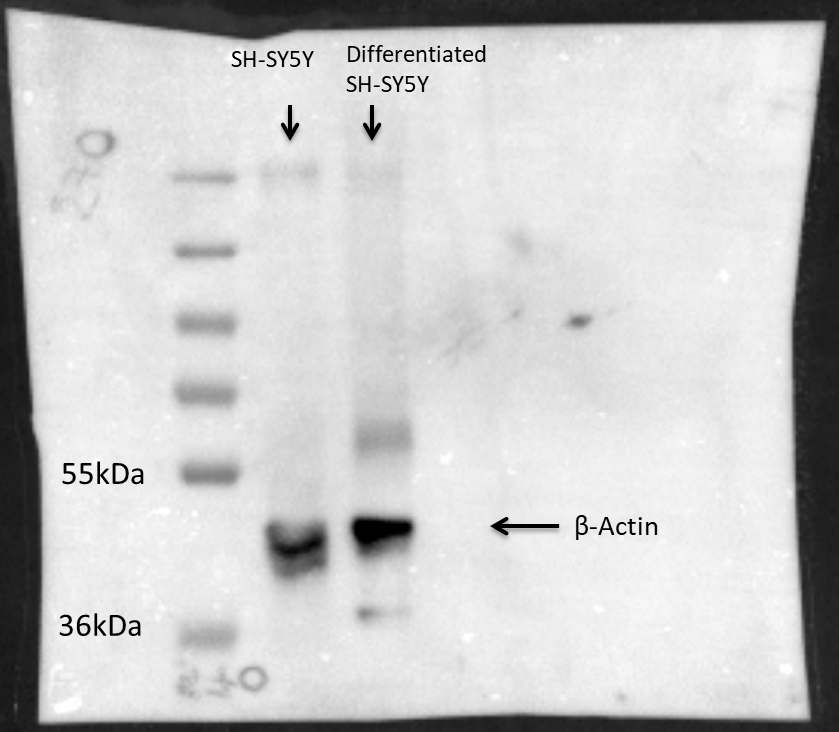

Supplement: Supplementary file 1 [file ijms-24-09486-s001.zip › Figure S2.tif]
